# Human organoid biofilm model for assessing antibiofilm activity of novel agents

**DOI:** 10.1038/s41522-020-00182-4

**Published:** 2021-01-25

**Authors:** Bing (Catherine) Wu, Evan F. Haney, Noushin Akhoundsadegh, Daniel Pletzer, Michael J. Trimble, Alwin E. Adriaans, Peter H. Nibbering, Robert E. W. Hancock

**Affiliations:** 1 Department of Microbiology and Immunology, University of British Columbia, Vancouver, BC Canada; 2 Department of Microbiology and Immunology, University of Otago, Dunedin, Otago New Zealand; 3 Department of Infectious Diseases, Leiden University Medical Center, Leiden, The Netherlands

**Keywords:** Biofilms, Antimicrobials

## Abstract

Bacterial biofilms cause 65% of all human infections and are highly resistant to antibiotic therapy but lack specific treatments. To provide a human organoid model for studying host-microbe interplay and enabling screening for novel antibiofilm agents, a human epidermis organoid model with robust methicillin-resistant *Staphylococcus aureus* (MRSA) USA300 and *Pseudomonas aeruginosa* PAO1 biofilm was developed. Treatment of 1-day and 3-day MRSA and PAO1 biofilms with antibiofilm peptide DJK-5 significantly and substantially reduced the bacterial burden. This model enabled the screening of synthetic host defense peptides, revealing their superior antibiofilm activity against MRSA compared to the antibiotic mupirocin. The model was extended to evaluate thermally wounded skin infected with MRSA biofilms resulting in increased bacterial load, cytotoxicity, and pro-inflammatory cytokine levels that were all reduced upon treatment with DJK-5. Combination treatment of DJK-5 with an anti-inflammatory peptide, 1002, further reduced cytotoxicity and skin inflammation.

## Introduction

There has been considerable discussion concerning the antibiotic resistance threat as resistance and multi-drug resistance rises and insufficient new antibiotics are being discovered^1^. Of similar or even greater concern are biofilm infections since not a single drug has been approved for use against such infections, despite the fact that biofilms represent 65% and 80% of all microbial and chronic human infections respectively^2,3^. Currently, the treatment of biofilm infections often involves surgical debridement and the use of a combination of antibiotics developed for free-swimming (planktonic) bacteria^4,5^. However, this is problematic because biofilms are adaptively multi-drug resistant, exhibiting inhibitory concentrations that are 10–1000 fold more resistant to virtually all conventional antibiotics^6^, and can rapidly recover from surgical debridement^4^. One enormous limitation in managing biofilm infections is the lack of convenient testing models due to the difficulty of recapitulating the clinical features of such infections^7^. There are no standardized in vitro biofilm tests like the minimal inhibitory concentration assays implemented by the Clinical and Laboratory Standards Institute and animal models of biofilm infections are often complex, of uncertain relevance, and often do not reflect human conditions^8^.

One example of biofilm infections is skin and soft-tissue infections (SSTIs) that afflicted 3.2 million people in the USA in 2012 for an aggregated cost of $15 billion^9^. *Staphylococcus aureus* and *Pseudomonas aeruginosa* biofilms are among the leading causes of SSTI^10^. *S. aureus* biofilms are commonly associated with chronic conditions such as atopic dermatitis^11^, diabetic foot ulcers^12^, and nosocomial infections in burn victims^13^, while *P. aeruginosa* biofilms cause major problems in burn and chronic wounds as well as implanted medical devices^14,15^. Current treatment regimens for SSTIs often include topical (e.g., mupirocin and retapamulin) and systemic (e.g., ciprofloxacin, cefazolin, and linezolid) use of broad-spectrum antibiotics^16,17^, which might contribute to the growing problem of antibiotic resistance while having poor efficacy. Therefore, novel therapeutics that directly target the bacteria within a biofilm would be advantageous for future management of biofilm infections.

Host defense peptides (HDPs) are evolutionarily conserved short polypeptides that exert biological effects ranging from direct antimicrobial activity to antibiofilm and immunomodulatory functions^18^. Our lab has taken the approach of screening synthetic peptides based on natural HDP templates, and selecting for potent antibiofilm sequences to combat antibiotic resistance^19,20^. Among the most effective peptides identified to date is a D-enantiomeric peptide, DJK-5, which exhibits broad-spectrum antibiofilm activity against bacterial pathogens^21^. It can eradicate oral biofilms^22^, inhibit *P. aeruginosa* biofilms in a lung epithelial model^23^, and reduce abscess size and bacterial burden in a murine cutaneous infection model^24^. These demonstrated antibiofilm effects make DJK-5 an attractive peptide candidate to test for efficacy against biofilm-associated skin infections.

Here, we describe an air–liquid interface skin epidermal model as an in vivo-like, humanized system adapted to study skin biofilm infections and developed to screen novel antibiofilm therapeutics. Furthermore, supplanting ethically challenging animal burn models^25^ with a burned skin methicillin-resistant *S. aureus* (MRSA) biofilm model developed here, enabled the study of biofilm infections and associated skin damage and inflammation.

## Results

### Characterization of bacterial biofilms on a skin surface

**Fig. 1:**
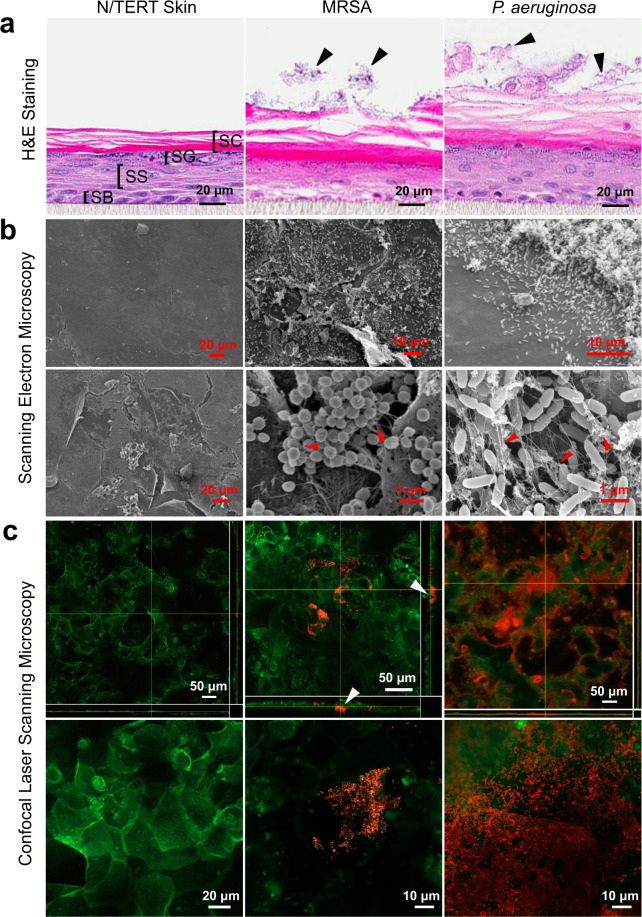
Microscopic characterization of MRSA and PAO1 biofilms on N/TERT skin. **a** Control skin and skin infected with one million MRSA (USA300-LAC) or PAO1 cells were visualized with H&E staining 24 h after seeding. Cross-sections of skin layers corresponding to the stratum corneum (SC), stratum granulosum (SG), stratum spinosum (SS), stratum basale (SB) were readily visible. MRSA and PAO1 biofilm on skin are indicated with arrows. **b** Control skin and 1-day MRSA-*lux* (MRSA SAP149) or PAO1-*lux* biofilm surface structures were imaged by SEM. Arrows indicate filamentous material resembling extracellular matrix interconnecting the MRSA and PAO1 biofilm. **c** Control skin or skin spotted with fluorescently tagged MRSA-FarRed or PAO1-mCherry was stained with CellMask^TM^ Green Plasma Membrane Stain and visualized using confocal microscopy 24 h after infection. Arrows indicate MRSA-FarRed bacteria clusters penetrated underneath the surface of SC. Each of the bottom images in (**c**) is a zoomed-in region of the orthographic projection shown above.

The morphology and architecture of 24 h bacterial skin biofilms were assessed by histological hematoxylin and eosin (H&E) staining, scanning electron microscopy, and confocal laser scanning microscopy. Histological analysis of the skin cross-section revealed a stratified skin structure 8–10 cells deep (Fig. 1a). Differentiated layers, including the stratum corneum, stratum granulosum, stratum spinosum, and the basal cell layer, were readily distinguished above the cell culture filter insert. When spotted with one million bacteria, clear aggregates could be observed on the skin surface 24 h after seeding, consistent with the formation of adhered biofilms^26^.

SEM imaging was performed to gain insights into the architecture of the skin surface structure. Untreated skin revealed some regions with smooth surfaces and other regions with rougher morphology, although we could not rule out the possibility that these were artifacts of fixation (Fig. 1b). The addition of Gram-positive MRSA to the skin resulted in small clusters of cells dispersed across the skin surface of the inoculation site. The application of Gram-negative *P. aeruginosa* resulted in a dense mat of adhered bacterial cells that completely covered the skin surface. High magnification images showed that MRSA and PAO1 biofilms were each inter-connected by thin filamentous extracellular matrices that we propose to be bacterial in nature.

To gain further insights into the organization of the skin biofilms and their penetration into the underlying layers, confocal microscopy was performed using engineered MRSA and PAO1 bacterial strains expressing red fluorescent proteins (Far-red fluorescent protein and mCherry respectively) coupled with a membrane-specific fluorescent dye, CellMask^TM^ Green Plasma Membrane Stain. This allowed for visual discrimination between cells within the biofilms and the membranes of the skin cells (Fig. 1c). Staining of uninfected skin with the membrane specific dye revealed layers of distinct and elongated keratinocytes at the surface of the differentiated skin. Based on the depth of staining, it appeared that the CellMask^TM^ Green dye only penetrated the top few cell layers of the skin corresponding to the upper layers of the stratum corneum seen in the H&E stained samples. Application of fluorescently labeled MRSA to the skin surface and growth for 24 h resulted in small microcolonies of bacteria present on the skin surface, as well as regions where bacterial aggregates had penetrated underneath the surface layer of epidermal cells (indicated by arrows). In contrast, fluorescently tagged PAO1 appeared as a dense mat of bacterial cells covering the entire surface of the skin.

### Effect of DJK-5 peptide treatment on skin associated MRSA biofilm

**Fig. 2:**
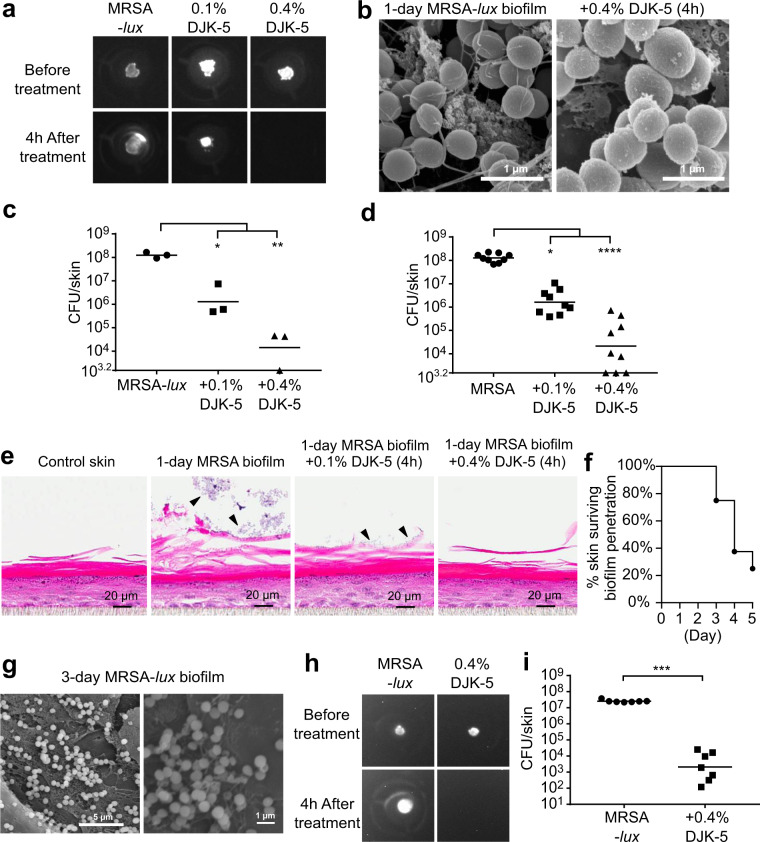
DJK-5 peptide reduced 1-day and 3-day pre-established MRSA biofilm on the skin surface. Skin biofilms were established by seeding one million MRSA (USA300-LAC) or MRSA-*lux* (MRSA SAP149) on top of the skin. Twenty-four hours post-infection, vehicle control or DJK-5 peptide at a dosage of 0.1% (30 μg) or 0.4% (120 μg) was administered on top of the skin. Four hours post-treatment, DJK-5 peptide reduced luminescence from skin colonized with MRSA-*lux* (**a**) and decreased total bacteria recovered from skin infected with MRSA-*lux* (**c**) and MRSA (**d**). Biofilm structures for MRSA-*lux* with or without DJK-5 treatment were visualized by SEM (**b**) and H&E staining (**e**). For the long-term study, MRSA-*lux* biofilm was imaged every 24 h after initial inoculation, (**f**) shows number of days that a confined biofilm can be maintained on top of the skin without bypassing the skin barrier resulting in bacterial growth in the culture media below the filter insert (raw data shown in Supplementary Figure 1a). MRSA-*lux* 3-day biofilm was visualized by SEM (**g**) and treated with 0.4% DJK-5 for 4 h, which reduced luminescence (**h**) and CFU recovered (**i**) compared to untreated samples. Statistical significance (*P ≤ 0.05; **P ≤ 0.01; ***P ≤ 0.001; ****P ≤ 0.0001) was determined using the Kruskal–Wallis test, Dunn’s multiple comparisons test (**c**, **d**), or the Mann–Whitney test (**i**). Geometric mean of CFU count from 3 to 9 biological replicates as indicated in (**c**, **d**, **i**).

To establish discrete, confined biofilms on top of the skin, MRSA or MRSA-*lux* strains were spotted on the surface of the epidermis. In the case of the MRSA-*lux* strain, 24 h after infection the area of biofilm colonization was visualized by imaging luminescence, which signifies actively metabolizing bacteria (Fig. 2a). Biofilms treated with vehicle control had no obvious change in luminescence intensity and often spread to the edge of the filter insert. Treatment with DJK-5 at a low dosage (0.1%, 30 μg total peptide) reduced the area of colonization, while a high dosage (0.4%, 120 μg) completely abolished the luminescence. Notably, the DJK-5 concentrations used here were less than the concentration of current topical antibiotics (e.g., 2% mupirocin or fusidic acid creams) used in clinical settings^27,28^. Since luminescence correlates with bacterial survival^29^, this suggests that DJK-5 was directly killing the bacterial cells within the biofilms. SEM images showed that, in contrast to untreated bacterial cells, MRSA-*lux* cocci treated with DJK-5 were enlarged and had rough surfaces sprinkled with small debris clusters, suggesting that DJK-5 treatment affected bacteria within the biofilm, causing damage to the bacterial cell wall and membrane. DJK-5 treatment also led to an apparent reduction in the string-like material, which we have concluded above might be bacterial biofilm matrix (Fig. 2b). It is not clear whether this reduction in biofilm matrix is due to a direct effect of DJK-5 on the matrix or peptide enhancement of natural biofilm dispersal^21^. DJK-5 treatment significantly reduced viable bacteria on the skin in a dose-dependent manner. The geometric mean of MRSA-*lux* bacteria decreased from 1.3 × 10^8^ CFU/skin for untreated samples to 1.6 × 10^6^ CFU/skin and 2.1 × 10^4^ CFU/skin for 0.1% DJK-5 and 0.4% DJK-5 treated skin samples respectively (Fig. 2c). Similar effects were seen for non-luminescent MRSA biofilms on skin surfaces treated with DJK-5 (Fig. 2d). To visualize skin and MRSA biofilm structures, H&E staining was performed on MRSA colonized skin samples four hours post-DJK-5 treatment (Fig. 2e). MRSA infected skin had clusters of bacteria attached to the surface, which also caused thickening and damage to the underlying stratum corneum. In peptide-treated samples, the bacteria on the skin surface were much less prominent and the damage to the stratum corneum was reduced (Fig. 2e). Although viable bacteria were recovered from biofilm treated with 0.4% DJK-5, minimal bacterial clusters in H&E stained samples may reflect an insensitivity of this method in visualizing biofilms.

Since the luminescence signal correlated with bacterial colony count recovered from each skin sample, this feature was used as a simple way of monitoring the progression of biofilm growth over time by imaging the luminescence from the biofilm every 24 h following the initial inoculation. We found that most MRSA-*lux* biofilms could be maintained on the skin surface for about 3 days without breaching the skin barrier which otherwise would result in overgrowth of bacteria in the media under the cell culture insert (Fig. 2f, raw data shown in Supplementary Fig 1a). Measurement of transepithelial electrical resistance (TEER) and FITC-dextran permeability confirmed that the skin organoids formed tight barriers, even after 1–3 days of biofilm formation, while after longer periods of incubation with biofilms, these assays confirmed disruption of skin integrity due to bacterial penetration (Supplementary Fig 2). Visualization of the 3-day MRSA-*lux* biofilm by SEM revealed similar aggregates of MRSA-*lux* cells to those observed with 1-day biofilms (Fig. 2g). Interestingly, DJK-5 was similarly effective at diminishing 3-day MRSA-*lux* biofilm as compared to 1-day biofilm. DJK-5 applied to the 3-day biofilms at a concentration of 0.4% successfully wiped out the bacterial luminescence (Fig. 2h) and significantly reduced bacterial load by 4 log orders of magnitude (Fig. 2i) within 4 h of treatment. Since the growth of biofilms was generally tolerated by the skin, it is worth noting that, at the end of the 3-day infection, MRSA-*lux* did not induce any increased cytotoxic effects or significant IL-1β and IL-8 release when compared to the uninfected control (Supplementary Fig 3a-c).

### Effect of DJK-5 peptide treatment on skin associated *P. aeruginosa* biofilms

**Fig. 3:**
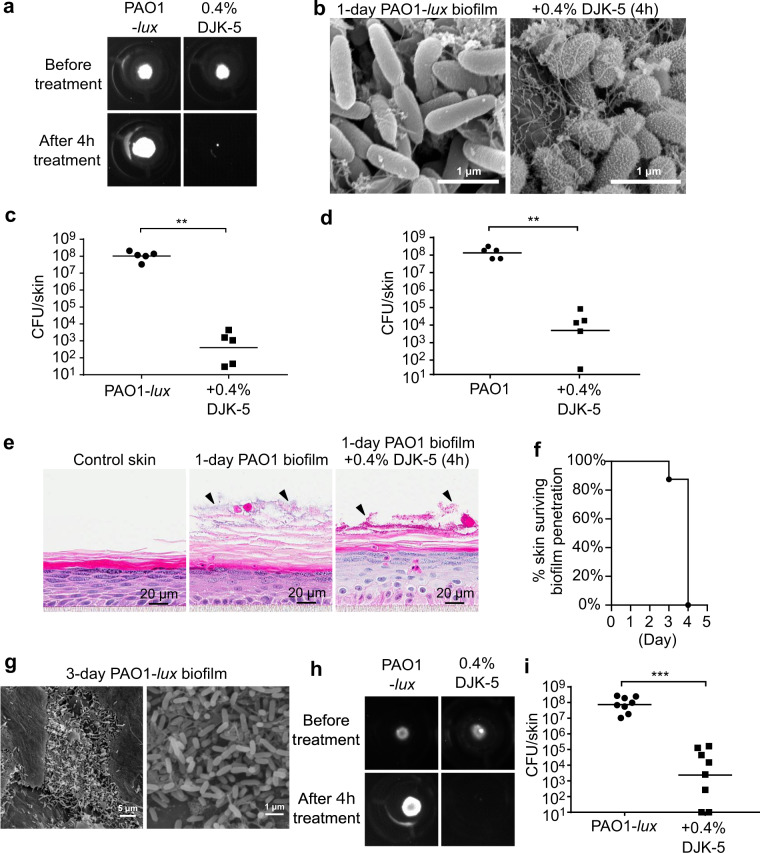
DJK5-peptide diminished *P. aeruginosa* PAO1 1-day and 3-day skin biofilm. Biofilms of PAO1 or its luminescent version (PAO1-*lux*) were seeded on the skin for 24 h then treated with 0.4% (120 µg) DJK5 peptide for 4 h. Luminescence signals from colonized PAO1-*lux* were imagined using the ChemiDoc Imaging System (**a**). Biofilm structures were visualized by SEM (**b**). Colony counts from skin samples infected with the luminescent (**c**) and non-luminescent (**d**) PAO1 were determined. Histological analysis of the biofilm structures was performed by H&E staining (**e**). The number of days that skin barrier withstood biofilm growth was monitored (raw data are shown in Supplementary Figure 1b) (**f**). PAO1-*lux* 3-day biofilm was imaged by SEM (**g**) and treated with 0.4% DJK-5 for 4 h, which reduced luminescence signals (**h**) and colony count (**i**). Statistical significance (**P ≤ 0.01; ***P ≤ 0.001) was determined using the Mann–Whitney test (**c**, **d**, **i**). Geometric mean of CFU count from 5 to 8 biological replicates as indicated in (**c**, **d**, **i**).

We further studied whether DJK-5 could eradicate Gram-negative *P. aeruginosa* PAO1 biofilms associated with N/TERT epidermal skin. Similar to MRSA, we initially used a luminescent version of PAO1 to visualize biofilm on the skin surface. We also tested DJK-5 at 0.4% only, since this concentration showed superior antibiofilm effects against MRSA. PAO1 biofilm treated with 0.4% DJK-5 for 4 h resulted in an evident reduction in the luminescence signal, whereas biofilms given vehicle control led to an increase in the area of colonization (Fig. 3a), possibly due to increased growth or additional lubrication/substrate to promote colony expansion/mobility. SEM analysis of the surface of DJK-5 treated PAO1 cells revealed numerous tube-like bleb structures and the shape of PAO1 cells transformed from rods to ovals, indicative of severe outer membrane damage, disruption in cell elongation, and a loss of shape maintenance^30^ (Fig. 3b). DJK-5 treatment exerted similar antibiofilm effects against the luminescent and untagged strains of PAO1. The geometric mean of recovered PAO1-*lux* bacteria declined from 8.9 × 10^7^ CFU/skin in control samples to 1.9 × 10^2^ CFU/skin in peptide treated skin (Fig. 3c), while non-luminescent PAO1 went down from 1.3 × 10^8^ CFU/skin to 4.9 × 10^3^ CFU/skin upon DJK-5 treatment (Fig. 3d). H&E staining revealed that colonization of the skin surface with PAO1 led to increased thickness in the stratum corneum and the appearance of PAO1 biofilm aggregates were mostly observed in the middle to upper layers of the stratum corneum (Fig. 3e). Following DJK-5 treatment, these clusters of PAO1 were diminished in size and this was accompanied by a reduction in stratum corneum thickness. Similar to MRSA, the skin barrier could endure the growth of PAO1 biofilm for about 3 days (Fig. 3f, raw data shown in Supplementary Fig 1b), and bacterial penetration coincided with substantially decreased TEER measurements and increased permeability to FITC-dextran (Supplementary Fig 2). The 3-day PAO1-*lux* biofilm structure was analyzed by SEM and similar clusters of PAO1-*lux* cells were observed (Fig. 3g). DJK-5 demonstrated a comparable antibiofilm efficacy on 3-day PAO1-*lux* biofilm, where peptide treatment wiped out luminescence signal after 4 h (Fig. 3h) and significantly decreased bacterial burden (Fig. 3i). Again no significant changes in toxicity and immune responses were triggered by PAO1-*lux* biofilms by day 3 (Supplementary Fig 3d-f).

### N/TERT skin biofilm model as a versatile platform for screening peptide activity

**Fig. 4:**
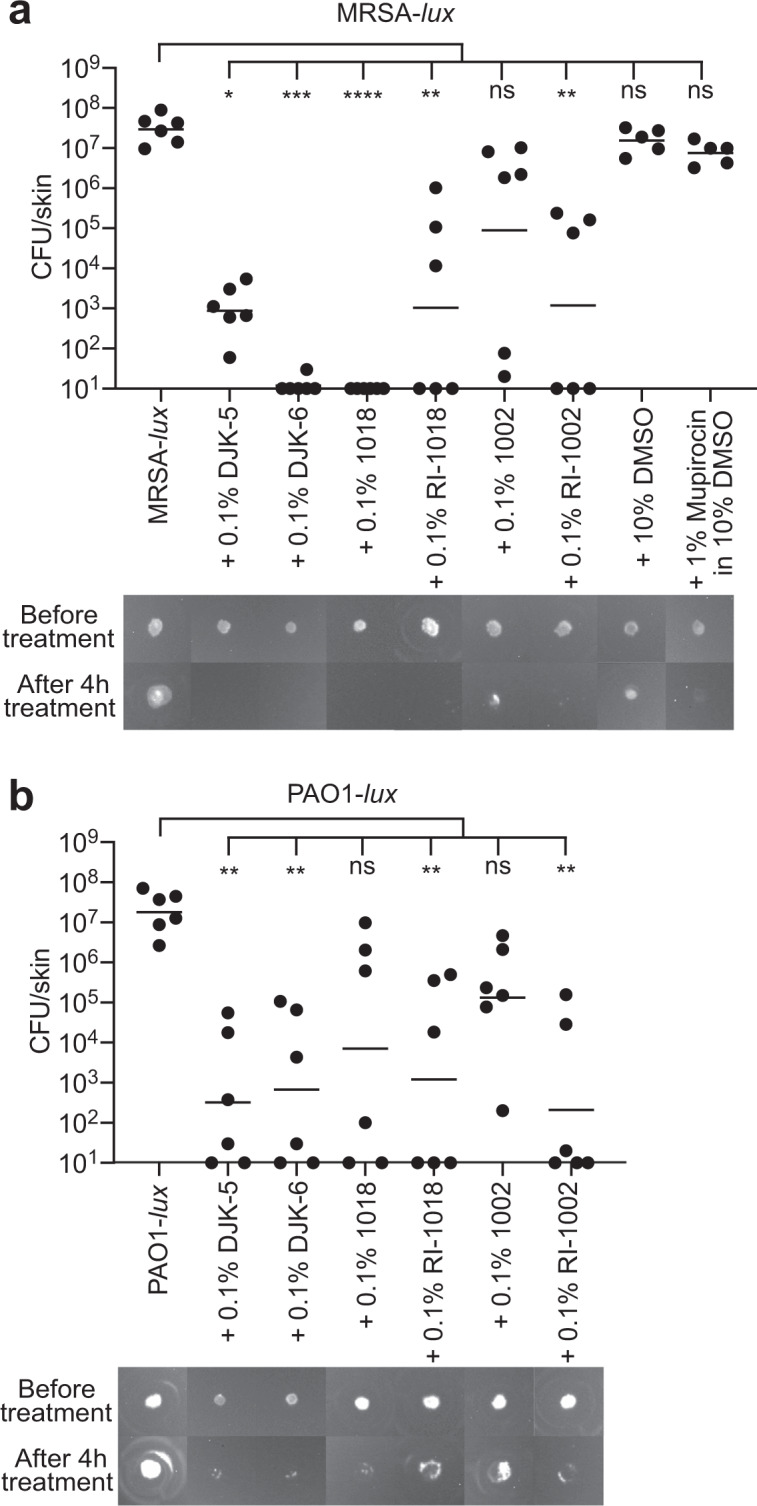
Differential antibiofilm activities of synthetic host defense peptides on epidermal skin biofilms. One day pre-established MRSA-*lux* (**a**) or PAO1-*lux* (**b**) biofilm were treated topically for 4 h with 0.1% of various synthetic HDPs, or 1% mupirocin dissolved in 10% DMSO, and the colony count recovered from each skin sample was determined. Luminescence signals from colonized MRSA-*lux* (**a**) and PAO1-*lux* (**b**) were imaged using the ChemiDoc Imaging System. Statistical significance (*P ≤ 0.05; **P ≤ 0.01; ***P ≤ 0.001; ****P ≤ 0.0001) comparing peptide treated skins to MRSA-*lux* or PAO1-*lux* control was performed using the Kruskal–Wallis test, Dunn’s multiple comparisons test. Geometric mean of CFU count from 5 to 6 biological replicates was indicated.

The one-day MRSA-*lux* (Fig. 4a) and PAO1-*lux* (Fig. 4b) skin biofilm system was used to screen the activity of several synthetic HDPs (sequences listed in Supplementary Table 1) at a peptide concentration of 0.1%. This approach revealed differential antibiofilm efficacies among the evaluated peptides that could be qualitatively assessed based on the luminescence (representative images shown in Fig. 4 bottom panel) or quantified by recovering CFUs following peptide treatment. All of the peptides except 1002 significantly reduced pre-established MRSA-*lux* (Fig. 4a) and PAO1-*lux* (Fig. 4b) biofilms in the context of this human organoid system within 4 h of treatment. Peptides DJK-5, RI-1018, and RI-1002 showed comparable antibiofilm activity against MRSA-*lux* and PAO1-*lux*, whereas DJK-6 and 1018 had superior effects in eradicating MRSA-*lux* biofilms. Notably, mupirocin, a Gram-positive-specific topical antibiotic often prescribed for superficial skin infections^31^, did not have appreciable impact on the bacterial load of Gram-positive MRSA-*lux* in the biofilm.

### DJK-5 reduced MRSA biofilm on thermally injured skin and altered cellular immune responses

**Fig. 5:**
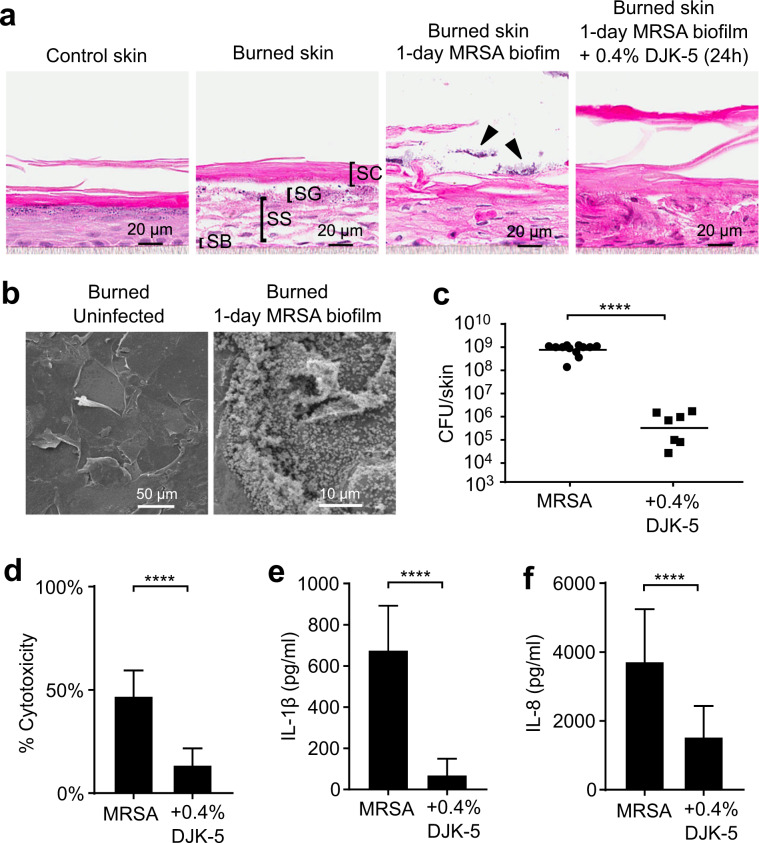
DJK-5 reduced MRSA biofilm, cytotoxicity, and pro-inflammatory cytokine production in a thermal burn skin model. Skin samples were subjected to thermal wounding for 4 s at 100 °C. One million MRSA (USA300-LAC) was then spotted on top of the burned skin and cultured for 24 h. Cross-sections of epidermal layers corresponding to the stratum corneum (SC), stratum granulosum (SG), stratum spinosum (SS) and stratum basale (SB) were visualized by H&E (arrows indicate MRSA biofilm) (**a**). (**b**) The burned skin and biofilm on top were visualized by SEM. (**c**) DJK-5 peptide in PBS at a dose of 0.4% (120 μg) or—as control—PBS was subsequently applied topically for 24 h. Samples were collected for bacterial counts (bars indicate geometric mean). (**d**) Culture supernatants below the filter inserts were used for measuring cytotoxicity by the lactate dehydrogenase assay. The amount of IL-1β (**e**) and IL-8 (**f**) in the media below the filter inserts was quantified by ELISA. Error bars indicate mean with SD in (**d**–**f**). Statistical significance was calculated using the Mann-Whitney test (**c**) or the student’s unpaired *t*-test (**d**–**f**) from 7 to 12 biological replicates (****P ≤ 0.0001).

Bacterial biofilms associated with damaged skin due to burns, physical injury, and disease associated conditions such as atopic dermatitis are difficult to treat as a result of defects in immune function and structural integrity of the skin^32,33^. To study the antibiofilm activity of DJK-5 peptide under conditions mimicking damaged skin, the skin model was “burned” using a digital soldering iron set to 100 °C for 4 s prior to establishing MRSA biofilm. Thermal challenge severely injured the epidermis, especially the stratum granulosum and the stratum spinosum layers, while the presence of MRSA biofilm further damaged the stratum corneum layer of the skin (Fig. 5a). Biofilm-infected burned skin that was treated with DJK-5 had a clear reduction in colonizing bacteria (Fig. 5a). SEM imaging suggested that burning of the skin did not cause major changes in the surface of the stratum corneum (Fig. 5b). MRSA biofilm colonized the surface and some gaps in between skin cracks (Fig. 5b). Topical treatment of the MRSA biofilm on the burned skin with 0.4% DJK-5 peptide for 24 h significantly reduced the bacterial burden on the burned skin, decreasing the bacterial load from 7.8 × 10^8^ CFU/skin to 3.2 × 10^5^ CFU/skin (Fig. 5c).

We further characterized the cellular immune responses of biofilm-infected burned skin by assaying the growth media underneath the cell culture inserts. MRSA colonization of burned skin triggered 47.0% cytosolic lactate dehydrogenase release, while DJK-5 treatment significantly decreased cytotoxicity induced by the MRSA biofilm to only 15% (Fig. 5d). MRSA infection also induced pro-inflammatory IL-1β and IL-8 production from thermally damaged skin, both of which were significantly suppressed by treatment with DJK-5 (Fig. 5e, f). Compared to control skin, skin that was thermally damaged and treated only with DJK-5 (no biofilm) caused only a small (~5%) increase in observed cytotoxicity (Supplementary Fig 4a) and slightly increased IL-8 production (Supplementary Fig 4b), suggesting that neither burning the skin nor peptide treatment, per se, triggered a large change in the overall cytotoxicity or immune response. Furthermore, normal skin with MRSA-*lux* or PAO1-*lux* biofilm established and treated with vehicle control (water) for 24 h (Supplementary Fig 4c–f), also only exhibited minimal cytotoxic effects (Supplementary Fig 4e) and an approximately 2-fold induction in IL-8 levels (Supplementary Fig 4f). Overall, these results demonstrate that burning of the skin increases the susceptibility and severity of the biofilm infection. We also attempted infecting the thermally damaged skin with strain PAO1, however, *Pseudomonas* consistently penetrated through the damaged skin resulting in growth in the skin culture medium under the cell culture insert within 24 h of inoculation (not shown).

### Combining antibiofilm peptide DJK-5 and anti-inflammatory peptide 1002

**Fig. 6:**
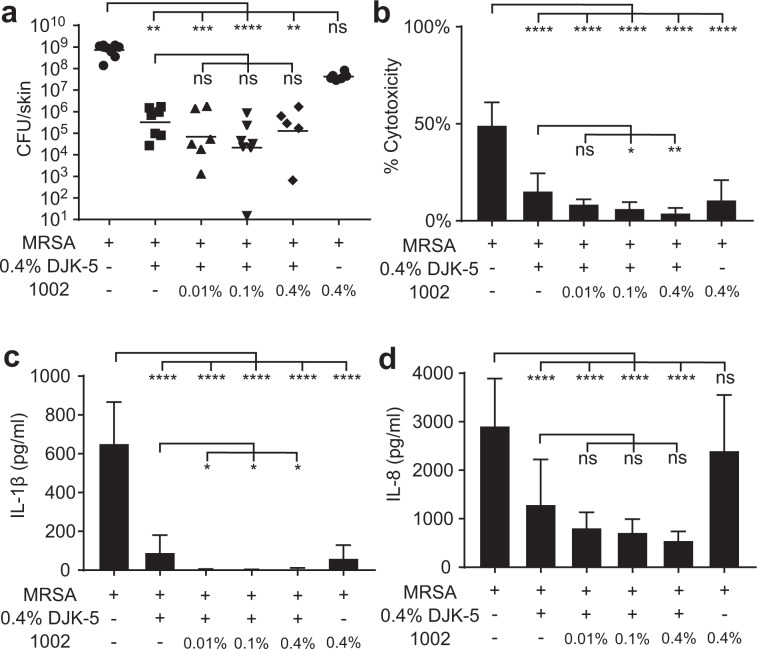
Peptides 1002 and DJK-5 combined treatment further dampened MRSA induced cytotoxicity and IL-1β production in the thermally damaged skin model. MRSA biofilms were established on top of thermally damaged skin. DJK-5 alone or in combination with 0.01%, 0.1%, or 0.4% (3 μg, 30 μg, or 120 μg) 1002 was added on top of skin biofilms for 24 h. Skin samples were harvested for bacterial counts (bars indicate geometric mean) (**a**), and culture supernatant was used to determine cytotoxicity by the lactate dehydrogenase assay (**b**) and anti-inflammatory activity by ELISA (**c**, **d**). Error bars indicate mean with SD in (**b**–**d**). Statistical significance from 5 to 12 biological replicates in each condition was determined using the Kruskal–Wallis test, Dunn’s multiple comparisons test (**a**) or one-way ANOVA, Dunnett’s multiple comparisons test (**b**–**d**) (*P ≤ 0.05; **P ≤ 0.01; ***P ≤ 0.001; ****P ≤ 0.0001).

Innate defense regulator peptide 1002 is a bactenecin derivative representing a class of synthetic HDPs capable of regulating host immune responses and dampening harmful inflammation^34^. Here, we tested a combination treatment of DJK-5 and 1002 against MRSA biofilm established on burned skin. The effect of 0.4% 1002 treatment alone on MRSA biofilms was marginal, resulting in only a 17-fold reduction in bacterial count compared to untreated biofilms (Fig. 6a). This was to be expected as this peptide exhibits relatively weak direct antimicrobial and antibiofilm effects in vitro^35^. Moreover, combining 0.4% DJK-5 treatment with 1002 at 0.01%, 0.1%, or 0.4% did not significantly enhance antibiofilm effects compared to DJK-5 treatment alone (Fig. 6a). However, despite its weak antibiofilm activity, 1002 treatment resulted in significant decreases in the cytotoxicity and pro-inflammatory IL-1β release from MRSA infected burned skin. When compared to DJK-5 single treatment, combination treatment with both DJK-5 and 1002 further reduced MRSA-induced cytotoxicity and IL-1β production (Fig. 6b, c). The combined treatment also caused a non-significant decrease in IL-8 release (Fig. 6d). Overall, these results demonstrate the potential of employing a multi-pronged strategy to combat recalcitrant skin biofilm infections using synthetic HDPs by simultaneously targeting both the pathogen and promoting a beneficial immune response.

## Discussion

There is increasing concern that the favorable results from in vitro screening and animal studies of novel drug compounds do not predict the outcomes of human clinical trails^36,37^. This problem drives the search for more informative experimental systems that are more representative of the in vivo conditions encountered during administration to man^38^. Organoids represent one of the most exciting tools for understanding disease pathology and testing novel drug toxicities and efficacies^38,39^. They have the added benefits of reducing the use of animals (which are often poor mimics to human systems) in pre-clinical testing and replacing in vivo infection models with an ethical alternative that better reflects human disease^40^.

Here, we described the adaptation of an air–liquid interface human skin model as an in vivo*-*like screening tool for novel agents against biofilm infections such as MRSA and *P. aeruginosa*. The use of luminescently tagged bacteria allowed for parallel activity comparison as well as monitoring the progression of the biofilm. Biofilms appeared as bacterial clusters upon H&E staining. We further discovered distinct biofilm morphologies in that MRSA biofilm appeared as small aggregates of cells whereas *P. aeruginosa* tended to form a more continuous dense mat on the skin surface. In addition, colonization by both species resulted in thickening of the stratum corneum and the epidermal skin barrier could tolerate the growth of these biofilms for about 3 days without an increase in cytotoxicity. Upon treatment with the antibiofilm peptide, DJK-5, we observed a membrane blebbing effect in PAO1 cells, which is a common phenomenon also seen in other Gram-negative bacteria when treated with host defense peptides. For example, time and concentration-dependent membrane blebbing has been observed in *Escherichia coli* cells treated with two peptides BP100 and PepR^41^. This is likely due to the polycationic nature of host defense peptides that can disrupt the anionic outer membrane of Gram-negative bacteria. Using this system, we also found that several peptides such as DJK-5, DJK-6, and 1018 have superior activity in eradicating MRSA biofilm, whereas mupirocin was almost inactive in this model. Consistent with this, MRSA strains that are sensitive to mupirocin treatment in their planktonic state or during very early biofilm formation can become highly mupirocin resistant in established biofilms^42,43^. For example, 2% mupirocin ointment, Bactroban, is largely inactive against 24 h biofilm of the mupirocin-sensitive MRSA (strain LUH14616)^41^. Indeed, sub-inhibitory concentrations of mupirocin have been shown to promote MRSA surface attachment and biofilm formation^44^. Together, these data underscore the inherent challenge in treating biofilm-associated infections in the clinic and highlight the value of the biofilm-based epidermal model in evaluating potential therapeutics for efficacy. In contrast to mupirocin, DJK-5 was equally effective against 3-day biofilms when compared to 1-day biofilms. This is likely because synthetic host defense peptides such as DJK-5, instead of targeting metabolically-active bacteria, function to promote the degradation of the stringent response mediator, guanosine tetraphosphate-(p)ppGpp, which is necessary for biofilm initiation and maintenance^45,46^. Therefore DJK-5 represents a viable therapeutic candidate for treating recalcitrant and long-term biofilm infections.

MRSA biofilm infection on burned skin caused enhanced cytotoxicity and inflammation, consistent with the clinical features of biofilm wound infections^47^. The bacterial burden recovered from thermally injured skin was nearly 10-fold higher as compared to skin biofilm in the absence of thermal damage. Since the epidermal barrier is crucial to confront and resist environmental stimuli (e.g., microbial colonization and changes in temperature, light, and water) and to maintain internal homeostasis^48^, the increase in bacterial burden could be due to an increase in MRSA colonization, increased spreading across the surface of the skin and/or penetration into the deeper layers of the tissue. Therefore, this suggests that thermal challenge negatively impacted the barrier properties of the skin, thereby increasing its susceptibility to MRSA infection. Consistent with this concept, in atopic dermatitis patients, *S. aureus* is able to more effectively penetrate lesional skin, when compared to non-lesional skin, as a result of impaired physical and antimicrobial barriers of the skin^49^. The host defense mechanism of the skin was activated due to skin damage and the bacterial burden exceeded the threshold that intact skin could otherwise tolerate. This is consistent with a previous study that found that MRSA infection enhanced the expression of IL-6, IL-8, and antimicrobial proteins human β-defensin (hBD)-2, hBD-3, and RNAse7 in thermally wounded skin when compared to control human skin^50^. In response to bacterial stimulation, the immune response of cells in the epidermis was found to be dependent on the depth of tissue being impacted by the infection; natural HDPs such as hBD-2 and hBD-3 were upregulated in the upper epidermis, whereas pro-inflammatory cytokines, such as IL-1β and IL-6, were mainly induced in the lower layers of the epidermis^51^. Therefore, the production of IL-8 and IL-1β in the burned-skin biofilm samples is an indication of MRSA penetrating deeper into the layers of the epidermal tissue, likely facilitated by increased tissue damage as a result of thermal wounding. Treatment with DJK-5, especially in combination with the anti-inflammatory peptide 1002, resulted in significant CFU reduction while also preventing excess tissue inflammation. This strategy is appealing since prolonged inflammation, accompanied by heavy bacterial burden, tissue breakdown, and necrosis, can adversely impact on chronic wounds since sustained inflammation creates a proteolytic environment that prevents the progression of wound healing into the proliferation phase^52,53^. It is worth mentioning that although the skin biofilm model can be used to screen antibiofilm and anti-inflammatory agents, there are certain limitations in accurately mimicking the human skin and skin wounds, including a lack of cellular diversity and no immune cells or blood circulation, which can impact the efficacy of antibiofilm peptides. To better understand the pathology of human wounded skin biofilm infections, this burned skin biofilm model could be coupled with ex vivo skin models^54^, which would in part add the complexity of the dermis and skin associated immune cells, and organs-on-a-chip technology^55^, which allow for the study of dynamic immune cell migration and blood flow processes.

In conclusion, we have demonstrated the utility of a biologically relevant, N/TERT cell-derived, skin biofilm model that can be used as a platform for testing the antibiofilm and immunomodulatory effects of synthetic HDPs. This system provides a reliable and robust alternative to animal models of skin infections and should help bridge the gap between the discoveries of novel antibiofilm agents to their clinical applications.

## Methods

### Synthetic peptides

Peptides used in this study are listed in Supplementary Table 1. The peptides were synthesized using solid-phase 9-fluorenylmethoxy carbonyl (Fmoc) chemistry and purified to ~95% using reverse-phase high-performance liquid chromatography. Peptide identity was confirmed by mass spectrometry. Stock solutions were prepared in sterile water and frozen at −20 °C until needed. On the day of the experiment, the peptide was diluted to the appropriate concentration in either water or sterile PBS. Aqueous stock peptide solutions had a pH of 5 and were subjected to no more than three freeze/thaw cycles.

### Reagents

Tryptic soy broth (TSB), Luria broth (LB), D-glucose, phosphate-buffered saline (PBS), Dulbecco’s PBS (DPBS), Keratinocyte-SFM medium, DMEM (high glucose, GlutaMAX™ Supplement, pyruvate), and Ham’s F-12 Nutrient Mix were purchased from ThermoFisher Scientific (Waltham, MA). DermaLife K Keratinocyte Complete Medium with LifeFactors was obtained from Lifeline Cell Technology (Oceanside, CA). CnT-Prime 3D Barrier Medium was purchased from CELLnTEC Advanced Cell Systems AG (Zurich, Switzerland). Gentamicin, lysostaphin trimethoprim, sucrose, glycerol, mupirocin, neutral-buffered formalin solution (10%), and various supplements for skin culture media including hydrocortisone, isoproterenol, bovine insulin, selenious acid, L-serine, L-carnitine, bovine serum albumin (BSA), palmitic acid, linoleic acid, and arachidonic acid were obtained from Sigma-Aldrich (St. Louis, MI).

### Bacterial strains and growth conditions

Bacterial strains used in this study include USA300-LAC^56^ (referred to as MRSA), and a luminescent MRSA strain SAP149^57^ (referred to as MRSA-*lux*), *Pseudomonas aeruginosa* strain PAO1^58^ and a luminescent strain of *P. aeruginosa*^59^ (PAO1-*lux*). Bacteria strains used for confocal microscopy imaging include *S. aureus* USA300-LAC transformed with a pKK22 plasmid expressing a far-red fluorescent protein (MRSA-FarRed), as well as *P. aeruginosa* PAO1 (PAO1-mCherry), transformed with an mCherry expressing plasmid, pMCh-23^60^. All *S. aureus* strains were grown overnight in TSB containing 1% D-glucose at 37 °C with shaking at 180 rpm, sub-cultured to mid-exponential growth phase in TSB 1% D-glucose. Bacteria were harvested and resuspended in sterile PBS to a concentration of 2 × 10^8^ CFU/ml before seeding onto skin surface. All *P. aeruginosa* strains were grown in LB under the same conditions as mentioned above, subcultured in LB medium, and resuspended in PBS at 2 × 10^8^ CFU/ml before seeding.

### Generation of fluorescently tagged MRSA and PAO1 strains

The 742-bp eqFP650 far-red fluorescent gene was excised from plasmid pSFRFPS1 via *Asc*I restriction sites and transferred onto *Asc*I-digested plasmid pKK22, yielding pKK22.eqFP650, and transformed into DH5αλpir. After confirmation of the correct orientation of the gene, the plasmid was transformed into *S. aureus* RN4220 as described below. Successful transformants were verified with a Synergy H1 96-well microtiter plate reader (BioTek, Winooski, VT) at wavelengths of 605 nm (ex) and 670 nm (em). The plasmid was re-isolated from RN4220 and transformed into USA300-LAC and transformants were verified via plasmid extraction and fluorescence measurements.

For plasmid transformation, *S. aureus* RN4220 or USA300-LAC was grown overnight in TSB. Overnight cultures were diluted to an OD of 0.5 in fresh TSB medium and incubated at 37 °C with shaking for 30 min. One ml was transferred into microcentrifuge tubes and harvested by centrifugation at 6200 g for 5 min. Preparation of electrocompetent cells was done, as previously described^61^. Briefly, cells were washed twice with an equal volume of autoclaved water, followed by one wash with 1/5 and one wash with 1/10 the volume 10% glycerol. All centrifugation steps were done at room temperature at 6200 g for 5 min. Subsequently, the final pellet was resuspended in 100 µl 500 mM sucrose and cells incubated with 2 µg DNA for 15 min at room temperature. DNA was transformed using a Gene Pulser Electroporator (Bio-Rad Laboratories, Berkeley CA) at 2.3 kV, 100 Ω, 25 μF. Cells were recovered in TSB for one hour at 37 °C and spread on TSA plates with 15 μg/ml Trimethoprim overnight at 37 °C. Successful plasmid transformants were confirmed via plasmid isolation (Qiagen, Venlo Netherlands) and restriction enzyme digest. Plasmids were isolated from overnight cultures that were first pelleted and re-suspended in buffer P1 (Qiagen) containing 10 μl Lysostaphin (5 mg/ml stock), and further incubated at 37 °C for 30 min before following the manufacturer’s instructions.

Plasmid pMCh-23 was transformed into electrocompetent *P. aeruginosa* PAO1, as previously described^62^. Briefly, cells were washed in 300 mM sucrose and electroporation of 500 ng plasmid DNA carried out at 2.5 kV, 25 μF, 200 Ω, using the Gene Pulser Electroporater. Transformants were selected on LB agar plates containing 50 µg/ml gentamicin. The mCherry expression in PAO1 was confirmed via fluorescence measurements at 580 nm (ex) and 610 nm (em).

### H&E staining

The filter insets containing N/TERT skin and MRSA biofilm were sandwiched between two foam biopsy pads (ThermoFisher Scientific) in a tissue embedding cassette (Sigma-Aldrich), fixed in 10% neutral-buffered formalin for 24 h, then transferred to 70% ethanol for storage. H&E staining was performed by Wax-it Histology Services Inc. (Vancouver, BC, Canada) and images were analyzed using the Aperio ImageScope software v12.4.0.5043 (Leica Biosystems, Wetzlar, Germany).

### Scanning Electron Microscopy (SEM)

Following MRSA-*lux* and PAO1-*lux* biofilm formation and DJK-5 treatment, the skin inserts were transferred to a fresh 12 well plate, washed twice with PBS, and submerged in 10% neutral-buffered formalin to fix the skin samples. The following day, the formalin was decanted and the fixed samples were washed in fresh buffer, dehydrated through a graded ethanol series (30%, 50%, 70%, 80%, 90%, 95%, 100%), and critically point dried (Tousimis Autosamdri 815B) over 24 h. Samples were sputter-coated (Cressington 208HR) with 10 nm AuPd. All SEM samples of 1-day old biofilms on skin were prepared twice while samples of 3-day old biofilms and burned skin were prepared once. Images shown are representative of the 3–10 images collected for each sample. SEM images in Fig. 1b (untreated skin, and MRSA-*lux* and PAO1-*lux* with 10 μm scale bars), 2 G, 3 G, and 5 were collected on a Hitachi S2600 Variable Pressure SEM (Hitachi, Ltd. Tokyo, Japan). SEM images in Fig. 1b (MRSA-*lux* and PAO1-*lux* with 1 μm scale bars), Figs. 2b and 3b were collected on a Hitachi S-4700 Field Emission SEM (Hitachi, Ltd. Tokyo, Japan).

### Confocal Laser Scanning Microscopy

Skin bacterial biofilms were grown for 24 h using either MRSA-FarRed or *P. aeruginosa* PAO1-mCherry. The samples were rinsed with DPBS and then stained for 10 min with CellMask^TM^ Green plasma membrane stain (Thermo Fisher Scientific) according to the manufacturer’s instructions. Samples were then fixed for 10 min in 10% formalin followed by three rinses in DPBS. All samples were stored at 4 °C and imaged within one week of harvesting. Confocal imaging was performed on a Zeiss LSM 800 Microscope equipped with a 20×/0.8 Plan-APOCHROMAT objective (Carl Zeiss Canada Ltd., Toronto, ON, Canada). Images were captured with the Zen software package (v2.6) and Z-stack images were analyzed in the Fiji software package^63^.

### N/TERT keratinocyte cell culture

N/TERT keratinocyte cells were kindly provided by Dr. Peter Nibbering (Leiden University Medical Center), Dr. Ivan Litvinov (McGill University), and Dr. Anna Mandinova (Massachusetts General Hospital), with permission from Dr. James Rheinwald (Harvard Medical School). N/TERT cells were maintained below 40% confluency in Keratinocyte-SFM medium supplemented with 25 μg/ml Bovine Pituitary Extract, 0.2 ng/ml human recombinant Epidermal Growth Factor 1–53 and 0.3 mM CaCl_2_ at 37 °C and 7.3% CO_2_. Culture media was refreshed every 2–3 days until ready for passage.

### The N/TERT epidermal skin

Skin models were established using a modification of described methods^64^. Briefly, 3 × 10^5^ N/TERT cells in 400 μl DermaLife K Keratinocyte Complete Medium with LifeFactors (5 μg/ml rh Insulin LifeFactor, 6 mM L-Glutamine LifeFactor, 1 μM Epinephrine LifeFactor, 5 μg/ml Apo-Transferrin LifeFactor, 0.5 ng/ml rh TGF-α LifeFactor, 0.4% Extract P LifeFactor, 100 ng/ml Hydrocortisone Hemisuccinate LifeFactor) were seeded onto each filter insert (ThinCert™ Cell culture insert, Greiner bio-one, Kremsmünster, Austria) in a 12-well ThinCert™ Plate (Greiner bio-one) holding 4.1 ml/well DermaLife K Keratinocyte Complete Medium with LifeFactors below each filter. Medium was refreshed every second day until N/TERT cells reached confluency in 3-4 days. Culture media both on top and below the filter were then switched to DMEM/Ham’s F-12/CnT-Prime 3D Barrier Media in a 3:1:4 ratio supplemented with 0.1 μg/ml hydrocortisone, 0.125 μg/ml isoproterenol, 0.25 μg/ml bovine insulin, 26.5 pM selenious acid, 5 mM L-serine, 5 μM L-carnitine, 1.6 mg/ml BSA, 25 μM palmitic acid, 15 μM linoleic acid and 7 μM arachidonic acid. Medium on top of the filters was removed the next day to allow air-exposure, which induced differentiation and stratification of the epidermis. After 2–3 days, linoleic acid concentration was increased to 30 μM. The skin samples were cultured for 10 days at air–liquid interface at 37 °C and 7.3% CO_2_ with medium being refreshed every 2–3 days.

### Bacterial biofilm and DJK-5 treatment

One million MRSA or PAO1, or luminescent MRSA-*lux* or PAO1-*lux*, or fluorescently-tagged MRSA-FarRed or PAO1-mCherry resuspended in PBS were seeded (5 μl of 2 × 10^8^ CFU/ml) in the center of the skin model, cultured at 37 °C and 7.3% CO_2_ to allow the establishment of bacterial biofilms. One day or three days after inoculation, 30 μl of 0.1% (1 mg/ml) or 0.4% (4 mg/ml) DJK-5 peptide was added on top of the biofilm for 4 h. To investigate how long the skin could endure biofilm growth, we monitored luminescence signals every 24 h after infection until luminescence was detected in the culture media underneath the skin, which indicated that the skin barrier had been breached. To visualize biofilms, skin samples seeded with luminescent bacteria were imaged using the ChemiDoc Imaging System (Bio-Rad). To quantify bacterial counts, skin samples, together with the filter inserts, were excised using a disposable scalpel (VWR, Radnor, PA), sonicated in 1.5 ml PBS, vortexed, serially diluted, and plated on LB agar plates. The cut-off of the Y-axis in each figure indicates the detection limit of CFU count while bars indicate the geometric mean of recovered CFU.

### MRSA USA 300 thermal wounding skin model

Thermal damage was created by burning 10-day air–liquid interface skins at 100 °C for 4 s with a digital soldering iron (FX888D, American Hakko Products, Inc., Santa Clarita, CA). The skin filter inserts were transferred to 12-well plates (Sigma-Aldrich) containing 800 μl/well fresh culture medium prior to bacterial infection. MRSA biofilm was established by seeding 2 × 10^6^ CFU in 5 μl PBS on top of the thermal damaged skin and cultured at 37 °C and 5% CO_2_ for 24 h. DJK-5 peptide (30 μl of 0.4%) alone or in combination with 0.01%, 0.1%, or 0.4% 1002 was administered on top of the pre-formed biofilm. Skin samples were collected 24 h post peptide treatment for colony count.

### Host response quantification

At the time of colony quantification, culture supernatants below the skin filter inserts were harvested for measuring cytotoxicity by the lactate dehydrogenase assay using a Cytotoxicity Detection Kit (Roche Diagnostics, Basel, Switzerland), according to the manufacturer’s instructions. Untreated skin samples or skin samples treated with 5% Triton X-100 were used as negative (0% toxicity) or positive (100% toxicity) control, respectively. Culture supernatants were also used to measure IL-1β and IL-8 production using ELISA kits from eBioscience (San Diego, CA).

### Reporting summary

Further information on experimental design is available in the Nature Research Reporting Summary linked to this paper.

## Supplementary information

Supplementary Information

Reporting Summary

## Data Availability

The authors declare that all data generated or analyzed in this study is included in the article or in the accompanying Supplementary Information file.
