# Supplementary material for: Human organoid biofilm model for assessing antibiofilm activity of novel agents
Source: NPJ Biofilms Microbiomes. 2021 Jan 25;7:8. doi: 10.1038/s41522-020-00182-4 (PMC7835231; doi:10.1038/s41522-020-00182-4)
Supplement: Supplementary file 1 — Supplementary Information [file 41522_2020_182_MOESM1_ESM.pdf]

Supplementary Figures

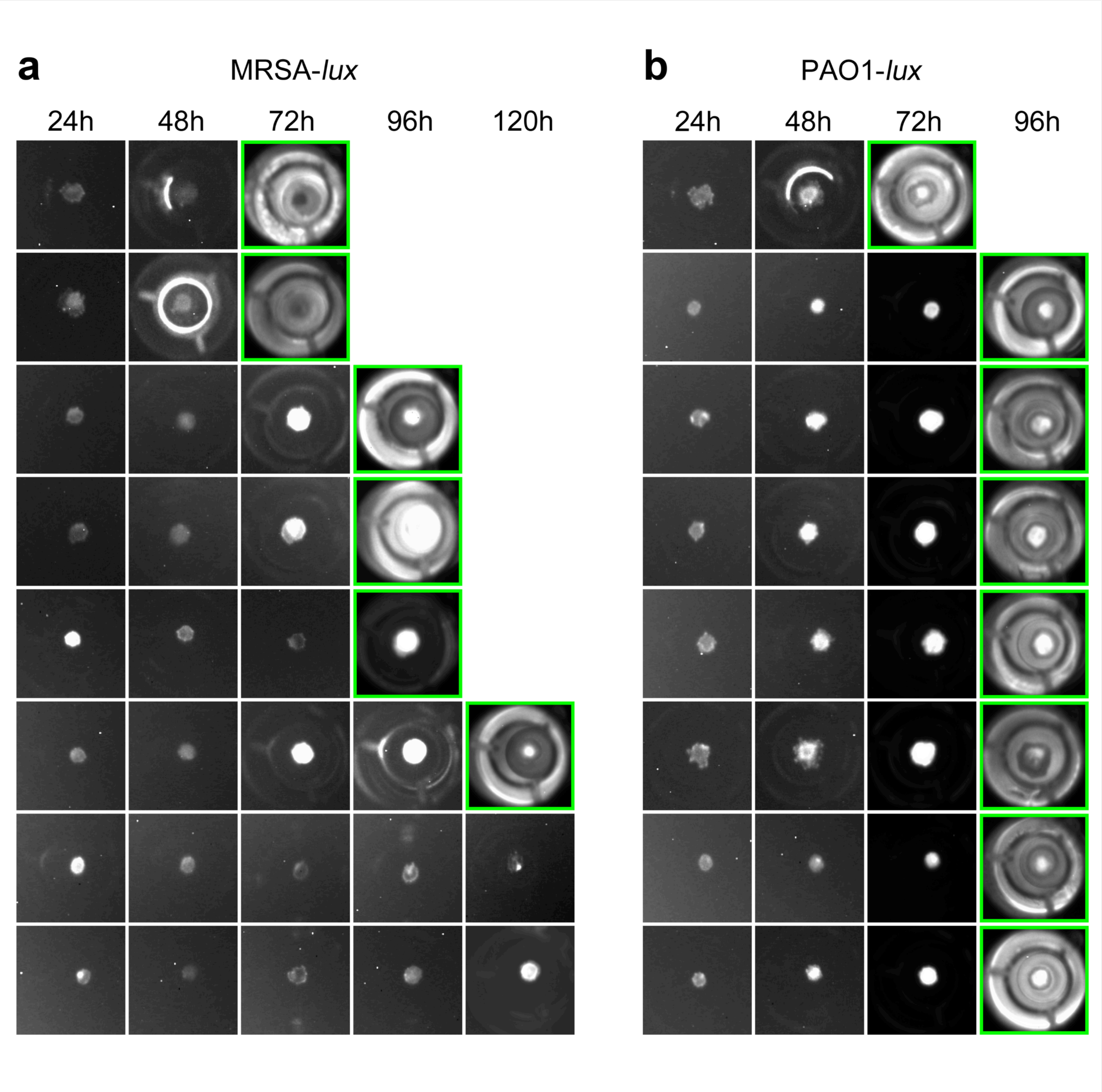

Supplementary Fig 1. The N/TERT skin could endure MRSA and PAO1 biofilm for about 3 days. MRSA-*lux* (**a**) or PAO1-*lux* (**b**) were seeded on top of skin and the resulting biofilms were imaged every 24 hours after seeding (n=8). Luminescence at the center of the filter insert indicated confined biofilm on the surface of the skin. In some cases, luminescence signals were detected in the growth media in the wells below the skin filter inserts (labeled with green outline), which indicated that bacteria penetrated through the skin barrier.

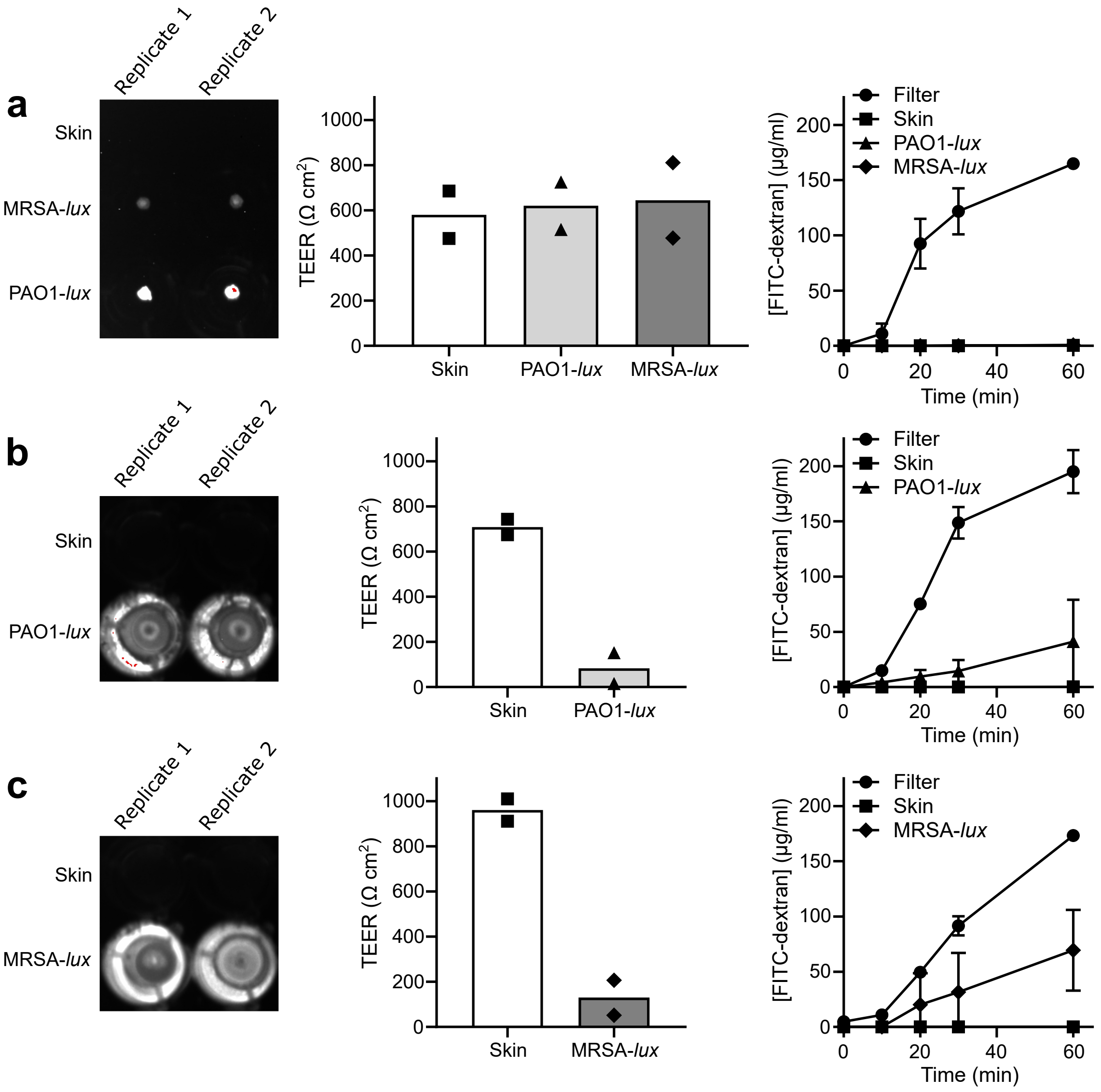

Supplementary Figure 2. Permeability of skin barrier in response to biofilm infection by PAO1-*lux* and MRSA-*lux*. Twenty four hours post infection (a), both MRSA-*lux* and PAO1-*lux* biofilms formed on the skin surface (left panel) but no large differences in TEER measurements (middle panel) or FITC-dextran permeability (right panel) were observed between samples, demonstrating that the skin barrier was intact in all samples. PAO1-*lux* bacteria penetrated through the skin after 48-hrs (b) resulting in reduced TEER values and increased permeability to FITC-dextran compared to uninfected skin samples. MRSA-*lux* bacteria penetrated the skin after 96-hrs (c), again resulting in reduced TEER and increased FITC-dextran permeability compared to uninfected skin.

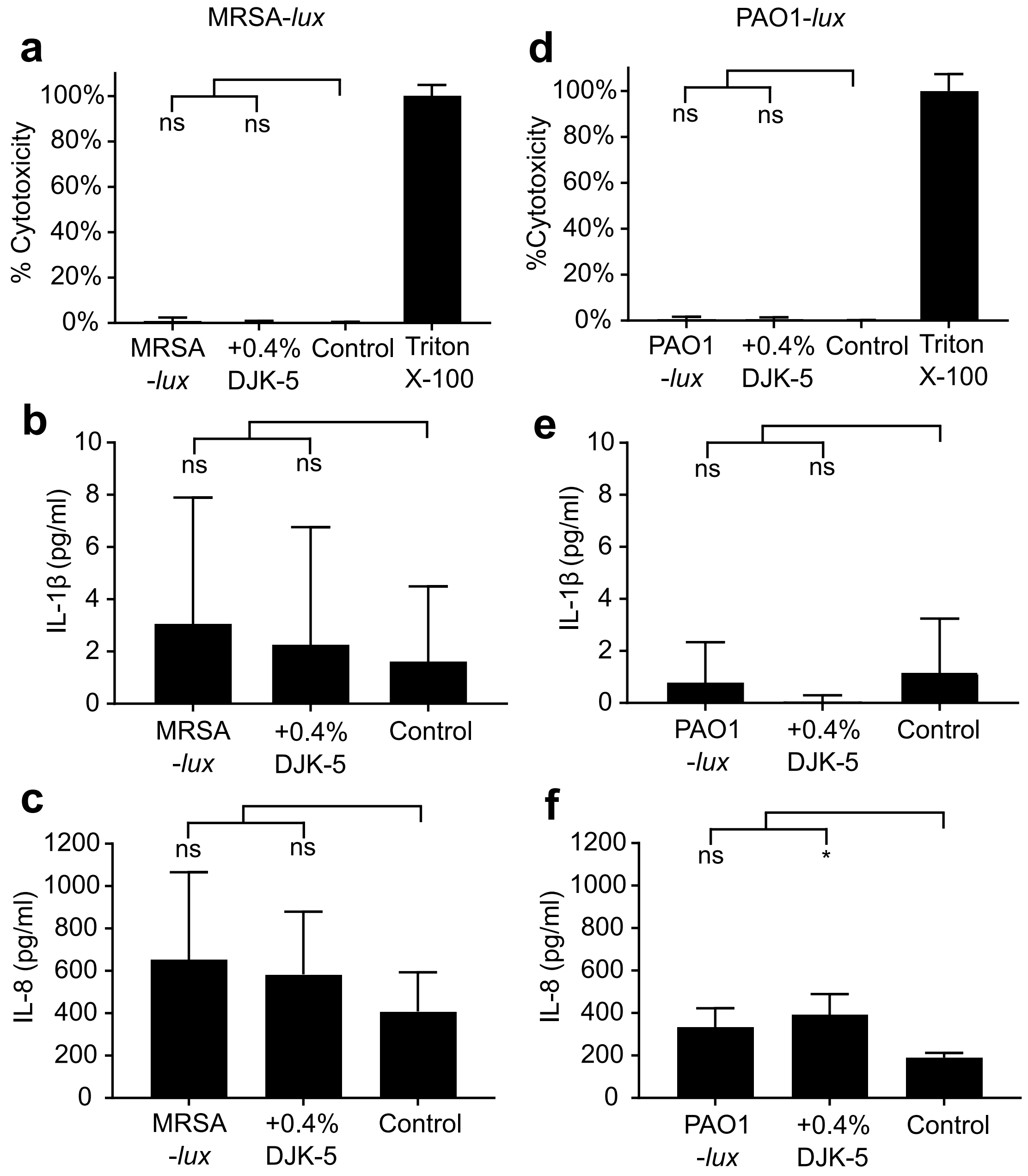

Supplementary Fig 3. N/TERT skin with 3-day MRSA and PAO1 biofilm had no cytotoxicity and based level cytokine production. MRSA-*lux* (**a, b, c**) and PAO1-*lux* (**d, e, f**) biofilm were established on top of epidermal skin for 3 days followed by 4-hour 0.4% DJK-5 peptide treatment. Culture supernatants below the skin were used for measuring cytotoxicity by the Lactate dehydrogenase assay. Untreated skin samples and skin samples treated with 5% Triton X-100 were used as negative (0% toxicity) and positive (100% toxicity) control, respectively (**a, d**). The amount of IL-1β (**b, e**) and IL-8 (**c, f**) in the supernatant was quantified by ELISA. Error bars indicate mean with SD (n=6). Statistical significance was performed using the One-way ANOVA, Dunnett's multiple comparisons test (* $P \leq 0.05$).

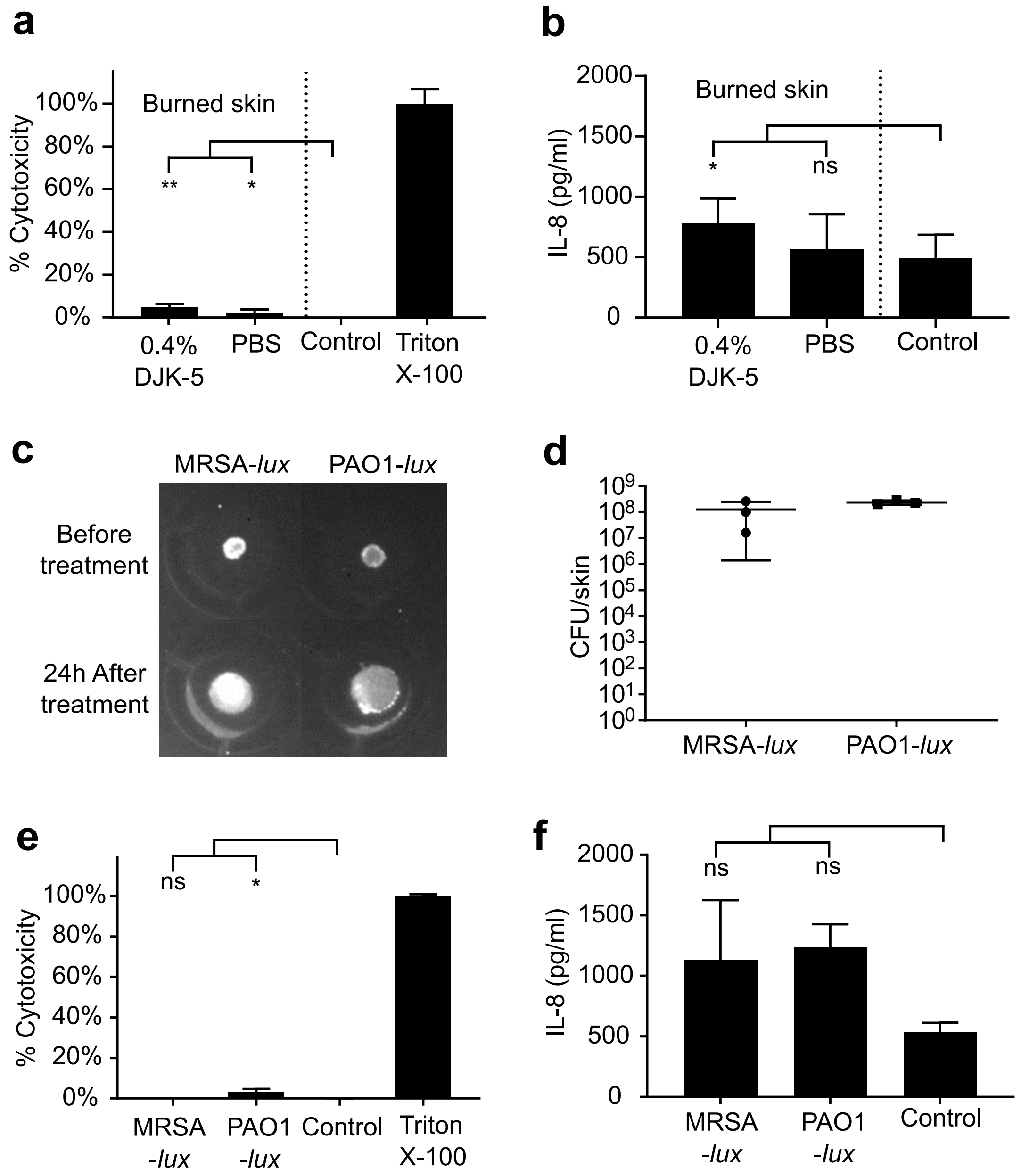

Supplementary Fig 4. Burned skin without infection and 2-day biofilm on skin without thermal damage had minimal changes in cytotoxicity and cytokine production compared to skin control without infection and thermal damage. Thermally damaged epidermal skin was mock infected with PBS for 24 hours, followed by 24 hours treatment with 0.4% DJK-5 or water (**a, b**). The cytotoxicity (**a**) and IL-8 production (**b**) from culture supernatant beneath skin was determined. One day pre-established MRSA-*lux* and PAO1-*lux* skin biofilm was treated with vehicle control-water for 24 hours, changes in luminescence signal (**c**), bacteria load (**d**), skin cytotoxicity (**e**) and IL-8 level (**f**) was quantified. Error bars indicate mean with SD (n=3). One-way ANOVA, Dunnett's multiple comparisons test (* $P \leq 0.05$; ** $P \leq 0.01$).

Supplementary Table

Supplementary Table 1. Synthetic host defense peptides investigated in this study

**Peptide**	**Enantiomeric Form (L or D)**	**Sequence (all peptides amidated)**	**Synthesized by**
DJK-5	D-Enantiomer	VQWRAIRVRVIR	CPC Scientific (Sunnyvale, CA)
DJK-6	D-Enantiomer	VQWRRIRVWVIR	Genscript (Piscataway, NJ)
L1018	L-enantiomer	VRLIVAVRIWRR	Genscript (Piscataway, NJ)
RI-1018	D-enantiomer	RRWIRVAVILRV	Genscript (Piscataway, NJ)
L1002	L-enantiomer	VQRWLIVWRIRK	Genscript (Piscataway, NJ)
RI-1002	D-enantiomer	KRIRWVILWRQV	Genscript (Piscataway, NJ)

Supplementary Methods

Skin Permeability Assays

Skin samples with or without bacteria were rinsed once on both the apical and basolateral side of the filter insert in phenol red free DMEM/F12 medium (ThermoFisher Scientific). The medium was then decanted and the filter inserts were transferred to a fresh well in a sterile 12-well plate. One milliliter of phenol free DMEM/F12 medium was added to the apical and basolateral side of the filter insert. Transepithelial electrical resistance (TEER) across the skin layer was measured using a Millicell ERS Volt-Ohm meter. The resistance across an empty filter membrane was subtracted from all sample measurements and TEER values (Ωcm^2) were calculated assuming a cell culture area corresponding to a filter area of 1.131 cm^2. Following TEER measurements, fluorescein isothiocyanate (FITC)-dextran 4000 (Sigma-Aldrich) was added to the medium on the apical side of the filter insert to a concentration of 500 μg/ml. The medium on the basolateral side of the filter insert was sampled (100 μl) at time intervals of 0, 10, 20, 30 and 60 minutes, replacing the equivalent volume removed with fresh medium at each time point. Samples were transferred to a black walled plate and FITC fluorescence was measured on a BioTek Synergy 2 Multi-Mode Microplate reader using an excitation wavelength of 487 nm and an emission wavelength of 528 nm. Data was recorded using optics at the top of the microplate at a read height of 7 mm. The concentration of FITC-dextran in each sample was determined based on a FITC-dextran standard curve that was prepared in parallel. All permeability assays were performed in duplicate.
