# Supplementary material for: Human organoid biofilm model for assessing antibiofilm activity of novel agents
Source: NPJ Biofilms Microbiomes. 2021 Jan 25;7:8. doi: 10.1038/s41522-020-00182-4 (PMC7835231; doi:10.1038/s41522-020-00182-4)
Supplement: Supplementary file 2 — Reporting Summary [file 41522_2020_182_MOESM2_ESM.pdf]

nature research

Corresponding author(s): Robert E.W. Hancock

Last updated by author(s): Dec 7th, 2020

Reporting Summary

Nature Research wishes to improve the reproducibility of the work that we publish. This form provides structure for consistency and transparency in reporting. For further information on Nature Research policies, see our Editorial Policies and the Editorial Policy Checklist.

Statistics

For all statistical analyses, confirm that the following items are present in the figure legend, table legend, main text, or Methods section.

n/a	Confirmed	
☐	☒	The exact sample size (n) for each experimental group/condition, given as a discrete number and unit of measurement
☐	☒	A statement on whether measurements were taken from distinct samples or whether the same sample was measured repeatedly
☐	☒	The statistical test(s) used AND whether they are one- or two-sided<br>*Only common tests should be described solely by name; describe more complex techniques in the Methods section.*
☒	☐	A description of all covariates tested
☐	☒	A description of any assumptions or corrections, such as tests of normality and adjustment for multiple comparisons
☐	☒	A full description of the statistical parameters including central tendency (e.g. means) or other basic estimates (e.g. regression coefficient) AND variation (e.g. standard deviation) or associated estimates of uncertainty (e.g. confidence intervals)
☐	☒	For null hypothesis testing, the test statistic (e.g. F, t, r) with confidence intervals, effect sizes, degrees of freedom and P value noted<br>*Give P values as exact values whenever suitable.*
☒	☐	For Bayesian analysis, information on the choice of priors and Markov chain Monte Carlo settings
☒	☐	For hierarchical and complex designs, identification of the appropriate level for tests and full reporting of outcomes
☒	☐	Estimates of effect sizes (e.g. Cohen's d, Pearson's r), indicating how they were calculated

Our web collection on statistics for biologists contains articles on many of the points above.

Software and code

Policy information about availability of computer code

Data collection	n/a
Data analysis	n/a

For manuscripts utilizing custom algorithms or software that are central to the research but not yet described in published literature, software must be made available to editors and reviewers. We strongly encourage code deposition in a community repository (e.g. GitHub). See the Nature Research guidelines for submitting code & software for further information.

Data

Policy information about availability of data

All manuscripts must include a data availability statement. This statement should provide the following information, where applicable:

- Accession codes, unique identifiers, or web links for publicly available datasets
- A list of figures that have associated raw data
- A description of any restrictions on data availability

The authors declare that all data generated or analysed in this study is included in the article or in the accompanying supplementary information file.

Field-specific reporting

Please select the one below that is the best fit for your research. If you are not sure, read the appropriate sections before making your selection.

☒ Life sciences ☐ Behavioural & social sciences ☐ Ecological, evolutionary & environmental sciences

For a reference copy of the document with all sections, see nature.com/documents/nr-reporting-summary-flat.pdf

Life sciences study design

All studies must disclose on these points even when the disclosure is negative.

Sample size	All SEM samples of 1-day old biofilms on skin were prepared twice while samples of 3-day old biofilms and burned skin were prepared once. Images shown are representative of the 3-10 images collected for each sample (This is stated in the Methods section of the manuscript). The TEER assay and FITC-dextran assay were preformed in duplicates (This is stated in the Supplementary Methods section). All other experimental conditions include at least 3 bio-replicates with the number specified clearly in the figure legends.
Data exclusions	No data were excluded from the analysis.
Replication	All SEM samples of 1-day old biofilms on skin were prepared twice while samples of 3-day old biofilms and burned skin were prepared once. Images shown are representative of the 3-10 images collected for each sample (This is stated in the Methods section of the manuscript). The TEER assay and FITC-dextran assay were preformed in duplicates (This is stated in the Supplementary Methods section). All other experimental conditions include at least 3 bio-replicates with the number specified clearly in the figure legends.
Randomization	The samples were assigned randomly to their treatment group.
Blinding	The investigators were not blinded to group allocation.

Reporting for specific materials, systems and methods

We require information from authors about some types of materials, experimental systems and methods used in many studies. Here, indicate whether each material, system or method listed is relevant to your study. If you are not sure if a list item applies to your research, read the appropriate section before selecting a response.

Materials & experimental systems

n/a	Involved in the study	
☐	☒	Antibodies
☐	☒	Eukaryotic cell lines
☒	☐	Palaeontology and archaeology
☒	☐	Animals and other organisms
☒	☐	Human research participants
☒	☐	Clinical data
☒	☐	Dual use research of concern

Methods

n/a	Involved in the study	
☒	☐	ChIP-seq
☒	☐	Flow cytometry
☒	☐	MRI-based neuroimaging

Antibodies

Antibodies used	Antibodies for ELISA were obtained from eBioscience, ThermoFisher Scientific<br>IL-8 Primary - Product number - AHC0982, lot TE2571641, clone 893A6G8<br>IL-8 Secondary - Product number - AHC0789, lot VG3030711, clone 790A28G2 1E3<br>IL1B Primary - Ref 14-7018-85, lot 1945067, clone CRM56<br>IL1B Secondary - Ref 13-7016-85, Lot 2124611, Clone CRM57
Validation	Human IL-8 antibody (AHC0982) is recommended by the manufacturer as a capture antibody in Sandwich ELISA applications with 9 publications (https://www.thermofisher.com/antibody/product/IL-8-CXCL8-Antibody-clone-893A6G8-Monoclonal/AHC0982).<br><br>Human IL1B (CRM56) antibody has been tested by the manufacturer as the capture antibody in a sandwich ELISA for analysis of human interleukin-1 beta in combination with the biotin CRM57) antibody for detection. It is cited in 11 publications (https://www.thermofisher.com/antibody/product/IL-1-beta-Antibody-clone-CRM56-Monoclonal/14-7018-85).

Eukaryotic cell lines

Policy information about cell lines

Cell line source(s)	N/TERT keratinocyte cells were kindly provided by Dr. Peter Nibbering (Leiden University Medical Center), Dr. Ivan Litvinov (McGill University) and Dr. Anna Mandinova (Massachusetts General Hospital), with permission from Dr. James Rheinwald (Harvard Medical School).
Authentication	This cell line was authenticated by typical keratinocyte morphology, consistent growth rate, and its ability to differentiate into structured skin. Also its response to various challenges is standard for this cell line. This cell line has been registered in the Expert Protein Analysis System (https://web.expasy.org/cellosaurus/CVCL_CW92) and published in several publications (1. Dickson, M. A., W. C. Hahn, Y. Ino, V. Ronfard, J. Y. Wu, R. A. Weinberg, D. N. Louis, F. P. Li, and J. G. Rheinwald. 2000. Human keratinocytes that express hTERT and also bypass a p16(INK4a)-enforced mechanism that limits life span become immortal yet retain normal growth and differentiation characteristics. Mol. Cell. Biol. 20: 1436–1447.; 2. Smits, J. P. H., H. Niehues, G. Rikken, I. M. J. J. van Vlijmen-Willems, G. W. H. J. F. van de Zande, P. L. J. M. Zeeuwen, J. Schalkwijk, and E. H. van den Bogaard. 2017. Immortalized N/TERT keratinocytes as an alternative cell source in 3D human epidermal models. Sci Rep 7: 11838.; 3. van Drongelen, V., M. O. Danso, A. Mulder, A. Mieremet, J. van Smeden, J. A. Bouwstra, and A. El Ghalbzouri. 2014. Barrier properties of an N/TERT-based human skin equivalent. Tissue Eng Part A 20: 3041–3049.)
Mycoplasma contamination	N/TERT keratinocyte cells were tested negative for mycoplasma contamination when providing to us.
Commonly misidentified lines (See ICLAC register)	No misidentified cell lines were used in this study.
